# Toxification of polycyclic aromatic hydrocarbons by commensal bacteria from human skin

**DOI:** 10.1007/s00204-017-1964-3

**Published:** 2017-04-04

**Authors:** Juliane Sowada, Lisa Lemoine, Karsten Schön, Christoph Hutzler, Andreas Luch, Tewes Tralau

**Affiliations:** Department of Chemical and Product Safety, German Federal Institute for Risk Assessment (BfR), Max-Dohrn-Strasse 8-10, 10589 Berlin, Germany

**Keywords:** Microbiome, Polycyclic aromatic hydrocarbons, PAHs, Benzo[*a*]pyrene, Toxification, Genotoxicity, Cytotoxicity

## Abstract

The ubiquitous occurrence of polycyclic aromatic hydrocarbons (PAHs) leads to constant human exposure at low levels. Toxicologically relevant are especially the high-molecular weight substances due to their (pro-)carcinogenic potential. Following ingestion or uptake, the eukaryotic phase I metabolism often activates these substances to become potent DNA binders, and unsurprisingly metabolism and DNA-adduct formation of model substances such as benzo[*a*]pyrene (B[*a*]P) are well studied. However, apart from being subjected to eukaryotic transformations PAHs are also carbon and energy sources for the myriads of commensal microbes inhabiting man’s every surface. Yet, we know little about the microbiome’s PAH-metabolism capacity and its potentially adverse impact on the human host. This study now shows that readily isolable skin commensals transform B[*a*]P into a range of highly cyto- and genotoxic metabolites that are excreted in toxicologically relevant concentrations during growth. The respective bacterial supernatants contain a mixture of established eukaryotic as well as hitherto unknown prokaryotic metabolites, the combination of which leads to an increased toxicity. Altogether we show that PAH metabolism of the microbiome has to be considered a potential hazard.

**Electronic supplementary material:**

The online version of this article (doi:10.1007/s00204-017-1964-3) contains supplementary material, which is available to authorised users.

## Introduction

With a surface of nearly 2 m^2^ the skin is our largest although toxicologically often less appreciated organ. Its protective function is essential, be it for mitigating exogenous impacts or by acting as physical and metabolic barrier against chemicals. Unsurprisingly, skin biology usually focuses on this very barrier function together with skin immunology (Veiga-Fernandes and Mucida 2016). This rather “eukaryo-centric” perspective has in recent years been complemented by the realisation that the skin also harbours a most diverse microbiome comprising more than 200 niche-specific genera (Clemente et al. 2012; Costello et al. 2009, 2013; Grice et al. 2009). However, the corresponding microbial populations are still primarily seen as a trait with immunological or olfactoric consequences at best (Clemente et al. 2012; El Aidy et al. 2012; Kerr et al. 2015; SanMiguel and Grice 2015).

Nevertheless, akin to the gut with its well-documented microbe–drug interactions the skin’s microbiome harbours a metabolic potential far exceeding that of its host. With a genome 100-fold the size of ours this includes the degradation and modification of ubiquitous xenobiotic substances such as polycyclic aromatic hydrocarbons (PAHs) (Tralau et al. 2015; Wallace and Redinbo 2013). Apart from constituting the majority of airborne pollutants (e.g. exhaust fumes, cigarette smoke, volcanic emissions), PAHs also occur in many consumer products, including cosmetics, tools and toys (Commins 1969; Lin et al. 2016; Morillo et al. 2007; Souza et al. 2016; Tarnow et al. 2016). Skin exposure is thus frequent with many of these substances being potentially harmful. Benzo[*a*]pyrene (B[*a*]P), for example, is subject to cytochrome P450-dependent monooxygenase (CYP)-mediated epoxidation in keratinocytes and other skin cells, leading to the formation of B[*a*]P-7,8-dihydrodiol-9,10-epoxide (Dekant 2009; Luch 2005; Shimada 2006). Unless eliminated via conjugation and excretion, the latter is a highly efficient DNA alkylating agent and as such a most potent carcinogen (Luch 2005; Luch and Baird 2010). At the same time, B[*a*]P is a potential substrate for skin bacteria, and although oxidative transformations are likely to occur, we know little about the corresponding metabolites or their toxicological potential for the human host (Sowada et al. 2014).

Previous data show the ability for commensal degradation of B[*a*]P and other PAHs not only to be widespread but to occur irrespective of age, gender or occupational pre-exposure (Sowada et al. 2014). Degradation was found to be partial as well as complete with the respective isolates presumably using different pathways for the degradation of B[*a*]P as sole source of carbon and energy. Microbial pathways need selective pressure to be maintained. The ready isolation of B[*a*]P-degrading organisms from skin therefore not only confirms permanent exposure but indicates biological relevance of this metabolism. We now report on the identification and toxicological characterisation of some of the corresponding excreted metabolites, several of them being cyto- and genotoxic.

## Materials and methods

### Chemicals and media

If not mentioned otherwise chemicals were purchased from Sigma-Aldrich (Taufkirchen, Germany) or Carl Roth (Karlsruhe, Germany) with the purity of chemicals used for enrichment cultures being greater than 98%. Media for cell lines and primary cells were sourced from PAN Biotech (Aidenbach, Germany) and Promocell (Heidelberg, Germany), respectively. Analytical reference substances (Table 1) were bought at the highest purity available from MRIGlobal Research (Kansas City, USA) and the PAH Research Institute (Igling-Holzhausen, Germany). Molecular reagents and kits were obtained from Qiagen (Hilden, Germany).

**Table 1:** List of reference substances used for metabolite identification and quantification

	rt/min	Quantifier m/z	Qualifier m/z
D_12_-B[*a*]P	18.36	406	316
B[*a*]P-3-ol	20.19	340	341
B[*a*]P-7-ol	19.90	340	341
B[*a*]P-8-ol	20.37	340	341
B[*a*]P-12-ol	19.54	340	341
B[*a*]P-1,6-dione	21.69	428	429
B[*a*]P-6,12-dione	20.51	428	429
B[*a*]P-11,12-dione	19.07	428	429
B[*a*]P-7,8-dione	21.57	428	429
B[*a*]P-9,10-diol	18.35	430	340
B[*a*]P-7,8-diol	21.35	430	340
B[*a*]P-4,5-diol	18.20	430	340
B[*a*]A	16.24	228	226
B[*a*]A-2-ol	17.87	316	301
B[*a*]A-4-ol	18.01	316	301
B[*a*]A-5-ol	17.76	316	301
B[*a*]A-9-ol	18.16	316	301
B[*a*]A-11-ol	17.53	316	301
B[*a*]A-3,4-diol	18.33	406	316
B[*a*]A-5,6-diol	16.65	406	316
B[*a*]A-8,9-diol	18.13	406	316
B[*a*]A-10,11-diol	17.75	406	316
Phe-4-CO_2_H	14.75	294	205
Pyrene	14.14	202	200
Anthracene	11.94	178	176

*B[a]P* benzo[*a*]pyrene, *B[a]A* benz[*a*]anthracene, *Phe* phenanthrene, *rt* retention time

### Bacterial growth

Bacterial growth was routinely estimated using optical density (OD, *λ* = 600 nm). Cultures for toxicity testing and metabolite analysis were set up as batch cultures at volumes of 150–250 ml using minimal growth medium with 100 µM B[*a*]P as sole source of carbon and energy (Sowada et al. 2014). Shake flasks were cultivated at 30 °C at 200 rpm with samples of 2 ml for supernatant testing and 2 × 25 ml for metabolite analysis taken every second day. Centrifugation for 10 min at 1000 rpm was used to separate the supernatant from cells as required and samples stored at −25 °C until further usage. Bacterial flask cultures were incubated for up to 4 weeks with non-inoculated mock cultures serving as negative controls for contaminations.

### Cell and tissue culture

The human immortal keratinocyte cell line HaCaT was cultivated in Dulbecco’s Modified Eagle’s Medium (DMEM) supplemented with 10% foetal calf serum (FCS) and 5 mg/ml penicillin/streptomycin at 37 °C in a humidified atmosphere with 5% CO_2_. Similar culture conditions were applied for HepG2 cells using Roswell Park Memorial Institute (RPMI) medium instead of full DMEM. Primary normal human epidermal keratinocytes (NHEK) from juvenile foreskin were obtained and isolated as described previously (Brinkmann et al. 2013).

### Cytotoxicity assays

Cytotoxicity was assayed in cellular assays based on glycolytic activity in at least three biological replicates. Assays were carried out using a 96-well microtitre format with 1 × 10^5^ cells per ml. Cells were subjected to substance treatment after an initial 24-h resting phase by adding 4 µl of bacterial supernatants or control substances, respectively. After 48 h of incubation, the assay was stopped by addition of 3-(4,5-dimethylthiazole-2-yl)-2,5-diphenyltetrazoliumbromide (MTT) at a final concentration of 500 µg/ml. Formation of formazan commenced for another 3 h. Following media removal and addition of DMSO (100 µl/well), the solubilised salt was then quantified in a “Genios Infinite M1000pro” microtitre plate reader (Tecan, Mainz-Kastel, Germany) based on its absorbance at *λ* = 595 nm.

### Single-cell electrophoresis assay (comet assay) and mutagenicity testing according to Ames

Analysis for genotoxic effects was done by using a modified comet assay as described previously (Brinkmann et al. 2013; Tarnow et al. 2016). Briefly, 1 × 10^5^ cells/ml were seeded into 24-well plates and left to rest for 24 h before being challenged with test substances as indicated. Methyl methanesulfonate (MMS, 80 µM) and B[*a*]P (2 mM) were routinely used as positive controls. While the first induces DNA damage directly, the latter requires oxidative activation and is thus indicative of the cellular phase I activity. Exposure to test substances lasted for 24 or 48 h. To increase the assay’s sensitivity DNA repair was inhibited by addition of 5 µg/ml aphidicolin being added after 2 h. For scoring, DNA was visualised using SYPBR Gold (Thermo Fisher Scientific, Darmstadt, Germany) and any comets quantified using the Image-Analyser Comet-Assay II software (Perceptive Instruments, Bury St Edmunds, UK). All experiments were performed as biological duplicates at least, scoring 50 cells per slide and using a call cut-off of 50 cells per slide. A concomitant bacterial reverse mutation assay (Ames test) was performed according to OECD guideline 471 as described previously by Buhrke et al. (Buhrke et al. 2013).

### Quantitative RT-PCR

Expression of selected CYP transcripts was analysed by quantitative RT-PCR using gene-specific primers (Table 2) and the “QuantiTect SYBR^®^ Green Kit” from Qiagen (Hilden, Germany). Cells were seeded into 6-well plates at a concentration of 1 × 10^5^ cells per ml and left to rest for 24 h before being subjected to substance treatment as indicated. Subsequent to cell harvest by trypsinisation extraction and reverse transcription of RNA was performed using “Omniscript Reverse Transcription Kit” (Qiagen, Hilden, Germany) as instructed by the manufacturer. In brief, 200 ng of mRNA was reversely transcribed using oligo-dT primers, followed by a gene-specific PCR for *CYP1A1* or the house-keeping control hypoxanthine–guanine phosphoribosyltransferase (*HPRT*), respectively. All experiments were performed as biological triplicates using an “Applied Biosystems 7500 Fast Real-time PCR System” (Thermo Fisher Scientific, Darmstadt, Germany) and relative transcript levels were calculated based on the c_T_ values as determined by the manufacturer’s “7500 Fast SDS Software” (Applied Biosystems, Thermo Fisher Scientific, Darmstadt, Germany).

**Table 2:** Primers used for quantitative RT-PCR

Primer	Sequence
CYP1A1 forward	5′-TCC AAG AGT CCA CCC TTC C-3′
CYP1A1 reverse	5′-AAG CAT GAT CAG TGT AGG GAT CT-3′
HPRT forward	5′-GTT CTG TGG CCA TCT GCT TAG-3′
HPRT reverse	5′-GCC CAA AGG GAA CTG ATA GTC-3′

### Gas chromatography coupled to mass spectrometry (GC-MS)

Metabolite analyses were performed using GC-MS. For each analysis, a total of 25 ml of bacterial supernatant were mixed with a mixture of internal standards (Table 3, 500 ng/ml in ethyl acetate) and subjected to extraction with 5 ml ethyl acetate. The extraction was repeated, collected, concentrated and transferred into fresh vials using methanol. The latter was subsequently removed by evaporation using a TurboVap system (Biotage, Uppsala, Sweden) at 40 °C and a gentle nitrogen air flow. The samples were then directly dissolved in 20 µl *N,O*-bis(trimethylsilyl)-trifluoracetamide (BSTFA) as derivatisation agent followed by silylation at 60 °C for 90 min and 120 rpm in a water bath. Sample injection used splitless mode technique to inject 1 µl of the silylated mixture into a gas chromatograph 6890 (Agilent Technologies, Waldronn, Germany) equipped with an HP-5MS capillary column (Agilent, 30 m × 0.25 mm × 0.25 µm) and coupled to a 5975 mass spectrometric detector (Agilent, sim/scan mode) operated in electron impact ionisation mode at 70 eV. The data acquisition was performed in combined mode of full-scan analysis and single-ion monitoring (SIM) mode, in order to achieve the highest sensitivity for analytes for which standard compounds are available while at the same time avoiding loss of information for unknown metabolites which might be not detected in the SIM mode. The column was operated in constant flow mode (1 ml/min) with helium 5.0 as carrier gas and an oven program ranging from 60 °C (for 1 min) to 320 °C (ramped at 15 °C/min, hold for 10 min). The transfer temperature was set at 295 °C. The injector was a cold injection system (Gerstel, Mülheim, Germany) starting at 45 °C, ramping to 300 °C at a rate of 12 °C/s. Metabolites were quantified using “GC/MSD ChemStation Software” (Agilent). Values from non-inoculated day 0 samples were used as blanks for background subtraction as necessary. Calibration series for all standards were prepared by dilution of stock solutions, for every sequence of real samples in *n*-hexane containing 5–500 ng/ml of the standard compounds and 500 ng/ml of the internal standards. Calibration curves were checked for linearity and used for quantification of the analytes.

**Table 3:** Internal standards used for verification of metabolite extraction and silylation, respectively

	Retention time/min	Quantifier m/z	Qualifier m/z
D_12_-B[*a*]A	16.12	240	120
9-Fluorenone	13.31	254	239
Pyrene-1-ol	16.30	290	275

## Results

### Human skin commensals excrete cytotoxic and genotoxic B[*a*]P metabolites

Aerobic bacterial degradation of PAHs such as B[*a*]P often involves dioxygenases (Brezna et al. 2003; Haritash and Kaushik 2009; Peng et al. 2008). The respective intermediates comprise epoxides as well as diols, aldehydes or carboxylic acids. These are similar but not necessarily identical to those known from eukaryotic metabolism (Brezna et al. 2006; Luch 2009; Peng et al. 2008). Yet, there are hardly any analytical or toxicological data available for metabolites that result from the bacterial degradation of B[*a*]P or other high-molecular weight PAHs.

**Fig. 1:**
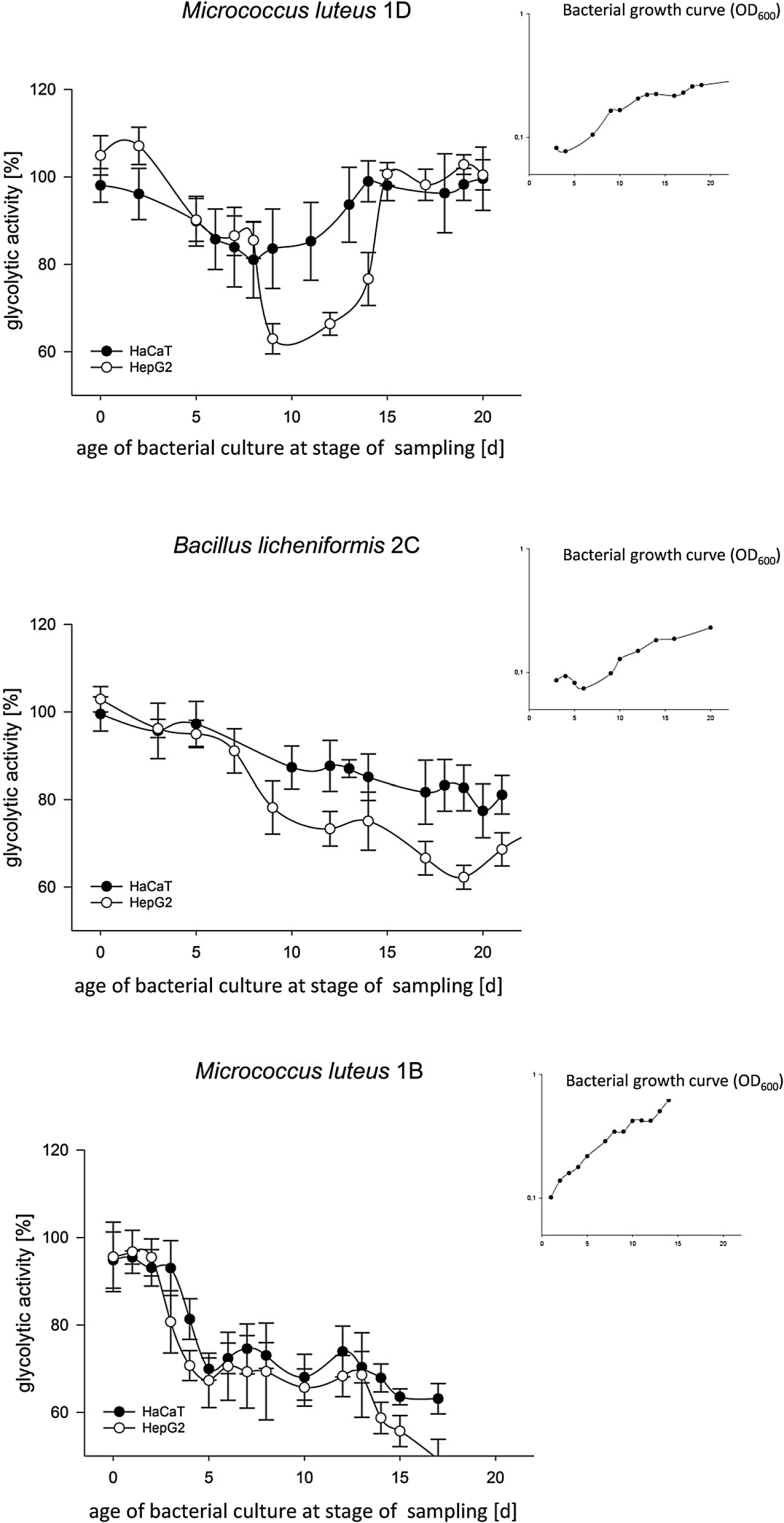
Cytotoxicity of metabolites as excreted by *M. luteus* 1D (**a**), *B. licheniformis* 2C (**b**) and *M. luteus* 1B (**c**) during growth on 100 µM B[*a*]P as sole source of carbon and energy. Batch cultures (inlets) were sampled as indicated and the supernatants added to cultures with HaCaT and HepG2 cells. Following 48 h of incubation cellular glycolytic activity was then recorded using an MTT assay as primary readout and microscopy for confirmation (data not shown, refer to suppl. Fig. S1). All data shown represent the mean of six biological replicates with *p* < 0.01. Negative controls comprised assays with bacterial minimal medium or cultures grown in lysogeny broth, all of which failed to induce signs of cytotoxicity (suppl. Fig. S2). Screens with NHEK cells produced similar results (data not shown, please refer to suppl. Fig. S3 for an exemplary plot)

Out of the 21 B[*a*]P-degrading isolates obtained previously, we selected three for further analytical and toxicological characterisation, namely *Micrococcus luteus* 1D, *Micrococcus luteus* 1B and *Bacillus licheniformis* 2C (Sowada et al. 2014). Their physiological parameters (i.e. substrate usage, growth rates and carbon yield) indicate these organisms to use different degradation pathways with *M. luteus* 1D being a total and *M. luteus* 1B or *B. licheniformis* 2C being partial degraders, respectively. Excretion of potentially cytotoxic metabolites was analysed in a cellular MTT assay and complementary microscopy by supplementing HaCaT cells, HepG2 cells or primary NHEK cells with supernatants from bacterial batch cultures grown on B[*a*]P as sole source of carbon and energy (Fig. 1).

The data show all strains to excrete cytotoxic metabolites, reducing cell viability down to 40%. Cytotoxic effects were recorded typically concomitant with the beginning of early exponential growth and were found to be transient for the complete degrader *M. luteus* 1D, while the other two isolates apparently accumulated cytotoxic end products. Effects were found to be more pronounced in HepG2 cells, probably as a result of additional phase I activation. Compared to skin cells, liver cell lines typically show higher levels of basal CYP expression. Yet RT-PCR nevertheless showed potential for further metabolic activation with extracts from the late exponential phase leading to a 70- to 100-fold induction of *CYP1A1* transcripts in HaCaT and NHEK cells, respectively (data not shown). Notably, this is in a range similar to the 122-fold induction observed with 2 mM B[*a*]P.

**Fig. 2:**
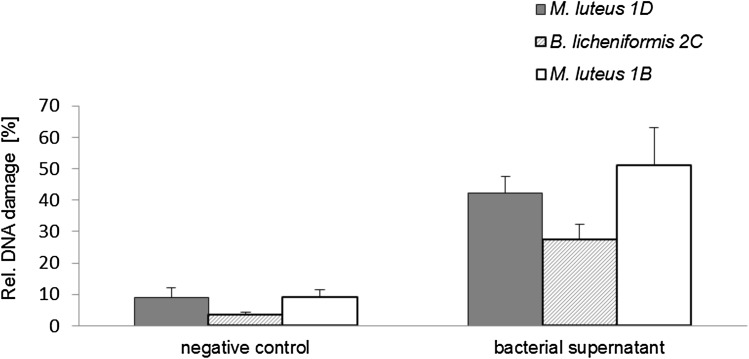
Genotoxicity of selected bacterial supernatants. Supernatants used had previously been tested positive for cytotoxicity before being assayed in a comet assay using HaCaT cells and 48 h of incubation. The results show levels of DNA damage relative to methyl methanesulfonate (MMS), using minimal medium without B[*a*]P as negative control. The data shown represent the mean of three biological replicates with *p* < 0.01. Similar levels of DNA damage were observed with NHEK cells or with B[*a*]P (data not shown)

In addition, a comet assay showed the respective supernatants to also feature genotoxic potential with the two *M. lutei* inducing higher amounts of DNA damage than *B. licheniformis* 2D (Fig. 2). Compared to the positive control, the two *Micrococci* induced 42 and 51% of relative DNA damage, contrastingly to the *Bacillus* with 27%. In addition, pre-screens showed the effects to be dose-dependent, again with the toxic potential ranging between 40 and 80% of what is seen with 1 mM B[*a*]P. However, at this late exponential phase B[*a*]P has already been used as growth substrate and the DNA damage is induced by the bacterial metabolites. The potential mutagenicity of the supernatants was confirmed further in a reverse mutation assay (Table S1). For *B. licheniformis* 2C, the Ames test recorded S9-dependent base-pair substitutions, while frame shifts and base-pair substitutions induced by *M. luteus* 1B did not require any metabolic activation.

### Metabolite identification and toxicity

**Fig. 3:**
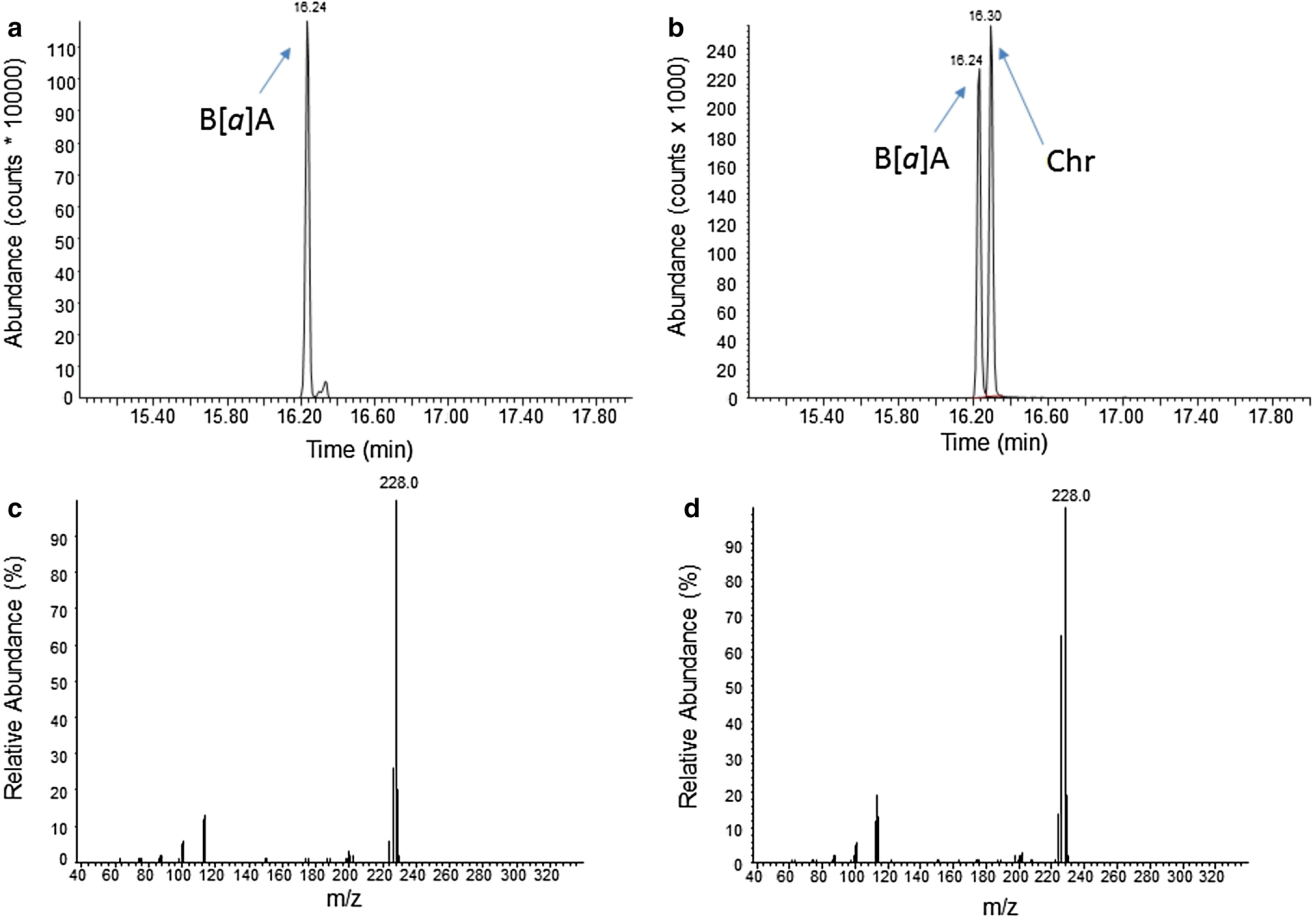
GC-MS chromatograms and corresponding full-scan mass spectra of benz[*a*]anthracene (B[*a*]A; m/z = 228; retention time = 16.24 min) as metabolite formed by *B. licheniformis* 2 C (**a, c**) and as synthetic compound (**b, d**). The reference chromatogram (**b**) also shows chrysene (Chr)

**Fig. 4:**
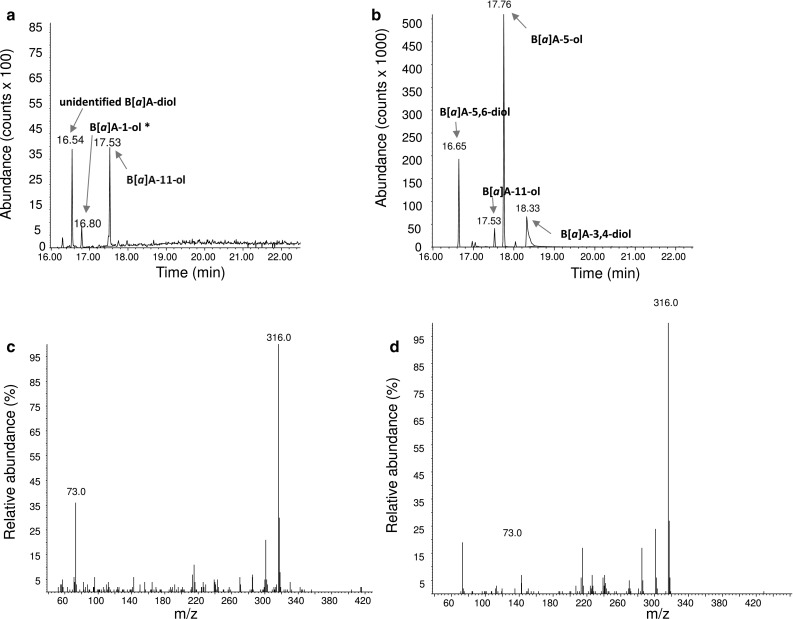
GC-MS chromatogram of B[*a*]A-11-ol (m/z = 316; retention time = 17.53 min) metabolically formed from B[*a*]P by *M. luteus* 1D (**a**), compared to the chromatogram of synthetic B[*a*]A-11-ol as part of a calibration mix (**b**). Also shown are the corresponding full-scan mass spectra for B[*a*]A-11-ol as metabolite (**c**) and as synthetic compound (**d**), respectively. *Qualitative identification based on chromatographic behaviour and properties as described by Grova et al. (2011)

Supernatant composition was subsequently analysed using GC-MS (Table 4). The data confirm all three isolates to have different metabolite patterns with the full degrader *M. luteus* 1D showing prominent excretion of B[*a*]P-1,6-dione, while the metabolite pattern of the partial degrader *M. luteus* 1B is dominated by B[*a*]P-7,8-dione and B[*a*]P-6,12-dione. Moreover, all isolates produced intermediates not found in eukaryotic metabolism. Again the corresponding patterns and peak excretions differed between the various organisms. Differences included, for example, varying concentrations of B[*a*]P-12-ol in all three isolates or the presence or absence of metabolites such as benz[*a*]anthracene (B[*a*]A), B[*a*]A-11-ol and phenanthrene (Phe)-4-CO_2_H in the supernatants of *B. licheniformis* 2C and *M. luteus* 1B. The identification of metabolically formed B[*a*]A in cultures of *B. licheniformis* 2C is illustrated in Fig. 3. Another unidentified B[*a*]A-diol was detected in the supernatant of *M. luteus* 1D (Fig. 4). In addition, the extracts contained several metabolites at low concentrations which could neither be qualified nor quantified unambiguously (data not shown, for an example please refer to Fig. 4a). Based on their chromatographic behaviour and properties, they are likely B[*a*]P-1-ol or B[*a*]P-2-ol, B[*a*]P-4-ol, or B[*a*]P-10-ol and B[*a*]A-1-ol (Grova et al. 2011).

**Table 4:** Quantities of metabolites identified in bacterial supernatants tested positive for toxicity (all values given are in nM)

	*M. luteus* 1D	*B. licheniformis* 2C	*M. luteus* 1B
B[*a*]P-9,10-diol	42		
B[*a*]P-1,6-dione	2050	56	277
B[*a*]P-6,12-dione			961
B[*a*]P-7,8-dione	41	220	>10,000^a^
B[*a*]P-3-ol	177	14	31 nM
B[*a*]P-7-ol	89	24	n.q
B[*a*]P-12-ol^*^	4		
B[*a*]A^*^	5	29	
B[*a*]A-11-ol^*^	26		
Phe-4-CO_2_H^*^	5		

The asterisk denotes metabolites not known to occur in eukaryotes

*B[a]P* benzo[*a*]pyrene, *B[a]A* benz[*a*]anthracene, *Phe* phenanthrene, *n.q*. not quantifiable

^a^Value of 16,948 nM is outside the linear range

**Fig. 5:**
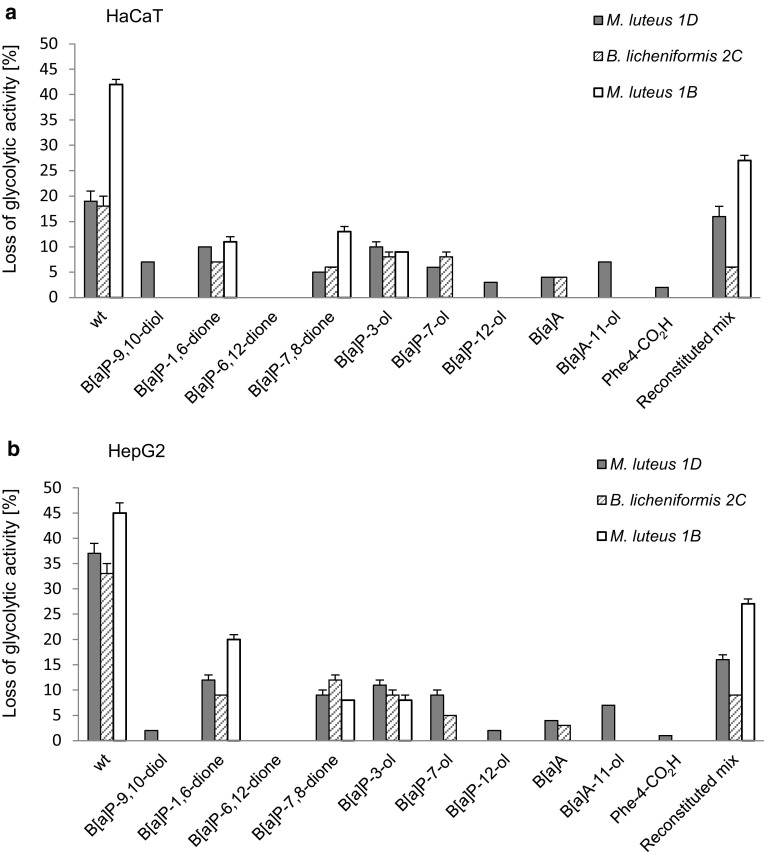
Cytotoxicity of bacterial B[*a*]P metabolites in HaCaT (**a**) or HepG2 (**b**) cells. An MTT assay was used to evaluate the cytotoxic potential (reduction of cellular glycolytic activity) of single metabolites and supernatants. Whereas “wt” denotes original culture supernatants of cultures grown on B[*a*]P-containing minimal medium, isolate-specific mixtures of identified metabolites in concentrations as excreted are referred to as “reconstituted”. Please refer to Table 4 for details. All data shown represent the mean of three biological replicates with *p* < 0.01

**Fig. 6:**
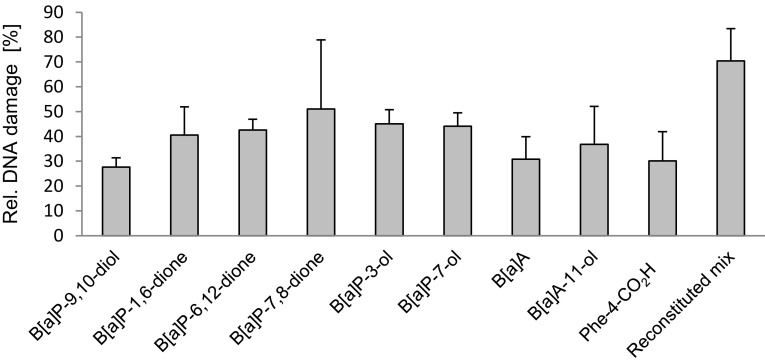
Relative genotoxicity of identified B[*a*]P-metabolites. The results show levels of relative DNA damage in HaCaT cells after 48 h of substance treatment. To maximise the sensitivity of the assay, metabolites were used at their highest excreted concentrations (please refer to Table 4) and as defined mixture thereof (“reconstituted mix”). The results show levels of DNA damage relative to MMS as positive control

The identified metabolites and selected combinations thereof were subsequently tested for cytotoxicity (Fig. 5). Generally, effects of the single substances on glycolytic activity were found to be weak. Only B[*a*]P-7,8-dione and B[*a*]P-1,6-dione and the two hydroxylated B[*a*]P metabolites showed some moderate effects. However, cytotoxicity was more pronounced following metabolite application as organism-specific mixture for *M. luteus* 1D or *M. luteus* 1B. Similarly, in the comet assay the genotoxic potential was more pronounced for the mixtures than for the single substances (Fig. 6).

## Discussion

Bacterial degradation of B[*a*]P has been observed before, although mostly in an environmental context (Cerniglia and Heitkamp 1990; Haritash and Kaushik 2009; Peng et al. 2008). The observation that B[*a*]P-degrading isolates can readily be isolated from human skin isolates thus came as a surprise (Sowada et al. 2014). Given the potential toxicity of oxidative B[*a*]P-metabolites, the wide occurrence of this phenomenon quickly leads to questions of metabolite identity and toxicity.

Analysing three of the previously obtained isolates, this study quantitatively identified a total of ten excreted metabolites (Table 4). Only six of these have previously been shown to also occur in eukaryotes (i.e. B[*a*]P-9,10-diol, B[*a*]P-1,6-dione, B[*a*]P-6,12-dione, B[*a*]P-7,8-dione, B[*a*]P-3-ol and B[*a*]P-7-ol), while B[*a*]P-12-ol, B[*a*]A, B[*a*]A-11-ol and Phe-4-CO_2_H seem to be specific for prokaryotic metabolism (Baird et al. 2005; Dekant 2009; Lin et al. 2016; Lorentzen and Ts’o 1977; Luch 2009; Shimada 2006; Souza et al. 2016; Sulc et al. 2016). Moreover, only three of these metabolites, namely B[*a*]P-1,6-dione, B[*a*]P-3-ol and B[*a*]P-7-ol, are also formed by the only B[*a*]P-degrading model organism, that is the environmental isolate *Mycobacterium vanbaalenii* PYR-1 (data not shown). The metabolite patterns of the three isolates confirmed the use of different degradation pathways, as also suggested by their substrate usage (Sowada et al. 2014). Befittingly the only three-ring metabolite (i.e. Phe-4-CO_2_) was detected in the supernatants of the fully degrading *M. luteus* 1D (Fig. S4), while the two partial degraders *B. licheniformis* 2C and *M. luteus* 1B only featured four- and five-ring systems. While this matches the expectations from the corresponding growth yields (i.e. 6.4, 1.6 and 2.9 g protein per mol carbon) (Sowada et al. 2014), it should be noted that the list of identified metabolites is not comprehensive. Although all major metabolites have been identified, the identification of some others remains ambiguous. This comprises the aforementioned B[*a*]A-diol from *M. luteus* 1D, as well as those that were only analysed qualitatively based on their chromatographic properties (i.e. B[*a*]P-1-ol or B[*a*]P-2-ol, B[*a*]P-4-ol, B[*a*]P-10-ol and B[*a*]A-1-ol) (Grova et al. 2011). Limiting factors are the transient metabolite formation together with low levels of excretion as well as chromatographic ambiguity. For example, distinction of some of the hydroxylated B[*a*]A derivatives from chrysenes requires the presence of the parent compound or extensive reference compound libraries, not all of which are readily available.

The supernatants of all three isolates were found to be cyto- as well as genotoxic. With metabolite concentrations in the low micromolar range, the overall cytotoxicity is comparable to that of the tumorigenic dibenzo[*a,l*]pyrene which features EC_50_-values of 0.8 and 0.4 µM in HaCaT and HepG2 cells, respectively (data not shown). Similarly, the DNA-damaging potential comes close to that of B[*a*]P or MMS, the latter of which being a typical inducer of direct DNA damage (Lundin et al. 2005). The accumulation of toxic metabolites for *B. licheniformis* 2C and *M. luteus* 1B is in concordance with their trait as partial degraders, while the full degrader *M. luteus* 1D only shows transient excretion. Although the higher cytotoxicity in HepG2 cells indicates a contributory role of eukaryotic phase I metabolism all effects were also clearly visible with HaCaT or primary NHEK cells, both of which only feature a limited metabolic competence (Bauer et al. 1995; Gotz et al. 2012; Oesch et al. 2014). The results therefore strongly indicate an inherent toxicity of the excreted metabolites even without further metabolism. Indeed B[*a*]P, B[*a*]P-7,8-dione and B[*a*]A are known for their cytotoxic potential with EC_50_ values in the mid-nanomolar to mid-micromolar range (Behrens et al. 2001; Burczynski and Penning 2000; Creusot et al. 2015; Kim et al. 1998; Shimada 2006). Yet, tests with the single substances at the concentrations equivalent to those in the corresponding supernatants found them only to be weakly toxic. This strongly indicates mixture effects, an observation also strengthened by the fact that the effect of the reconstituted supernatant of *M. luteus* 1D was close to that observed with the actual supernatants. Likewise genotoxicity could not be attributed to one particular analyte but appeared to be result of co-exposure. This is supported further by the Ames test, which indicated mutagenicity for some of the supernatants but not for single substances. This comes at little surprise as the Ames is known to be less sensitive particularly for substances with low solubility (Lah et al. 2008; Wu et al. 2009). Meanwhile when used at their peak concentrations, the toxicity of the overall metabolite mixture in the comet assay came close to that of the original bacterial supernatants.

This is the first time that the skin microbiome’s metabolism of PAHs such as B[*a*]P and its toxicological potential has been analysed. The data clearly show the metabolites formed to be cyto- and genotoxic at the concentrations excreted. Skin migration and penetration of high- and mid-molecular weight PAHs is strongly size-dependent and can reach up to 30% for four- and five-ring systems (Hutzler, personal communication; Paschke et al. 2014). Given the similarity of these substances to the bacterial metabolites, it is likely that similar values apply. Genes involved in bacterial B[*a*]P-degradation can be detected *in situ* as can transcripts of the 16s-RNA genes of the organisms involved (with copy numbers of up to 20,000 copies/cm^2^) (data not shown). In this context, exposure of the human host to bacterial B[*a*]P metabolites as potential carcinogens becomes only a question of B[*a*]P availability and concurrent microbial or eukaryotic metabolism. The toxicological impact of the microbiome’s metabolism hence clearly deserves more attention in the future.

## Electronic supplementary material

Below is the link to the electronic supplementary material.

Supplementary material 1 (DOCX 570 KB)
