# Supplementary material for: Toxification of polycyclic aromatic hydrocarbons by commensal bacteria from human skin
Source: Arch Toxicol. 2017 Apr 4;91(6):2331–41. doi: 10.1007/s00204-017-1964-3 (PMC5429354; doi:10.1007/s00204-017-1964-3)
Supplement: Supplementary file 1 — Supplementary material 1 (DOCX 570 KB) [file 204_2017_1964_MOESM1_ESM.docx]

**Sowada *et al*, supplementary material**


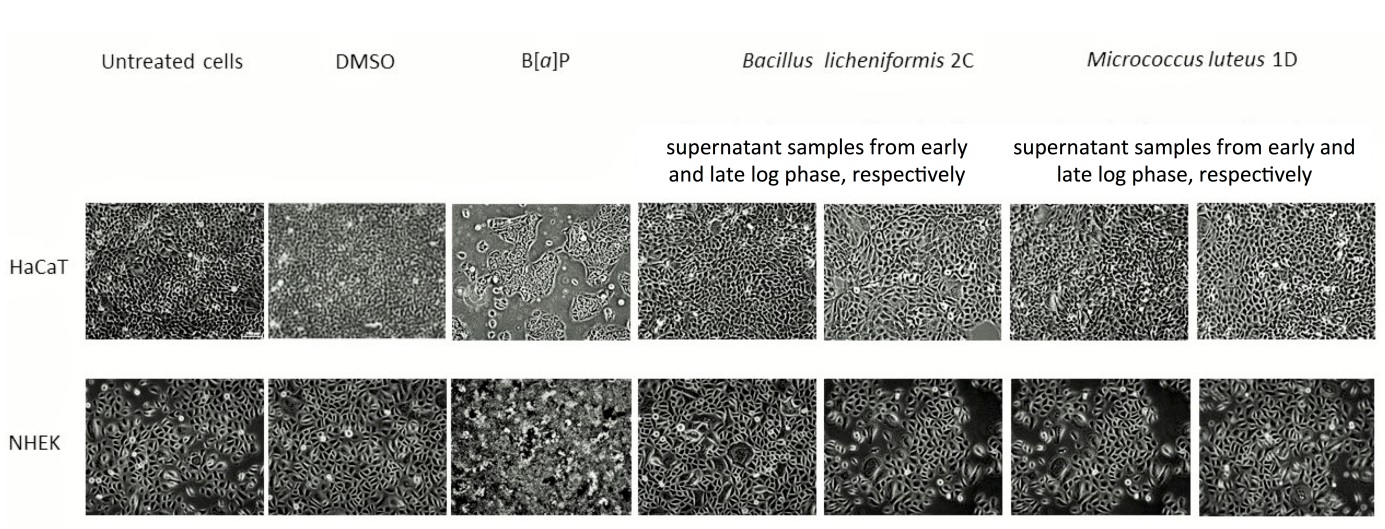


**Fig. S1.** Selected images of HaCaT and NHEK cells after treatment with bacterial supernatants as indicated. Cytotoxicity led to slower growth and pronounced differences in morphology, especially for HaCaT cells. Abbreviations: DMSO, dimethyl sulfoxide; B[*a*]P, benzo[*a*]pyrene.

**a**


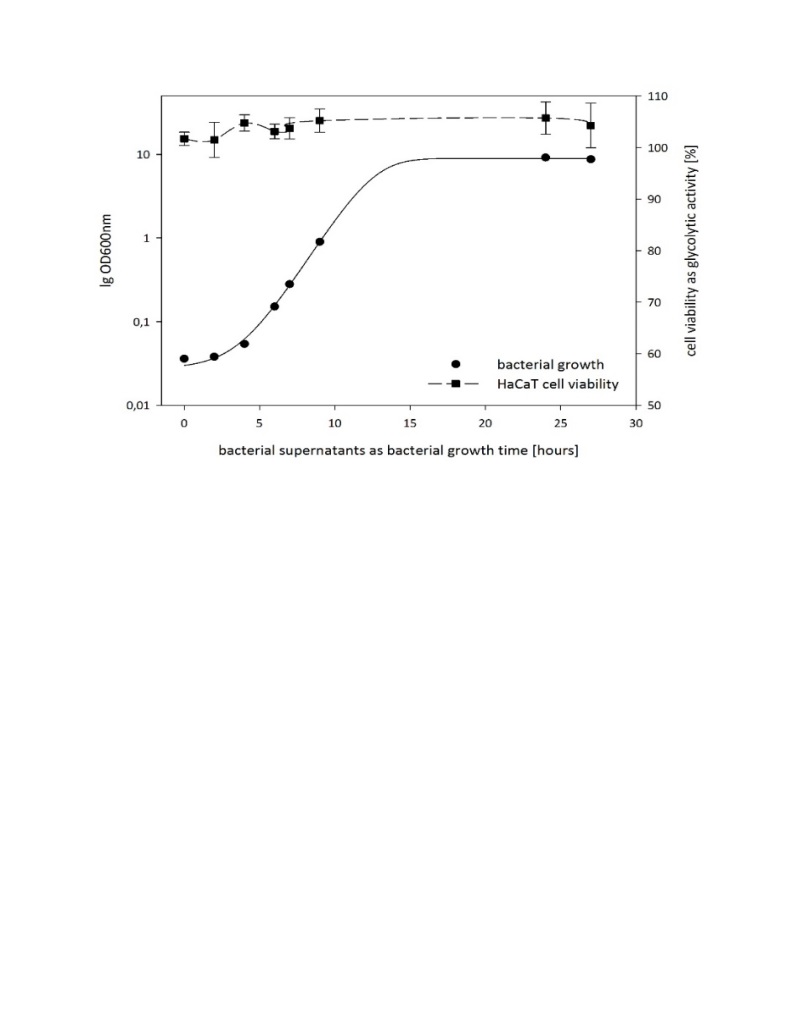


**b**


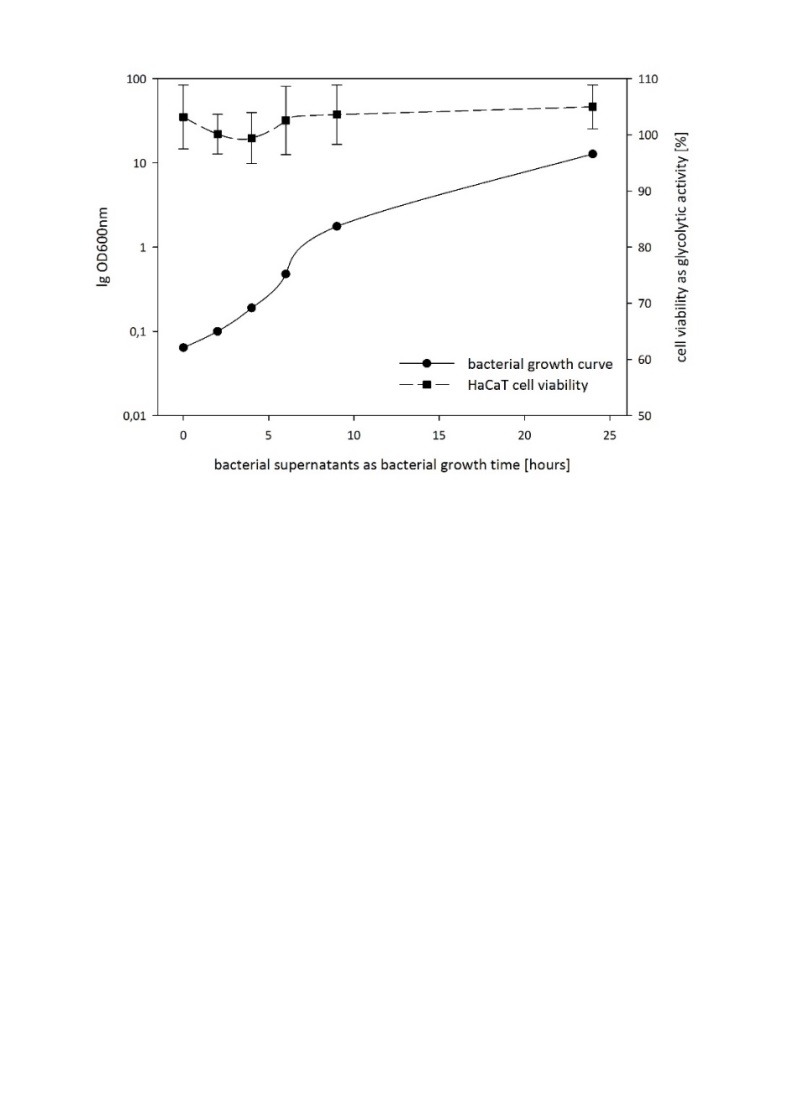


**c**


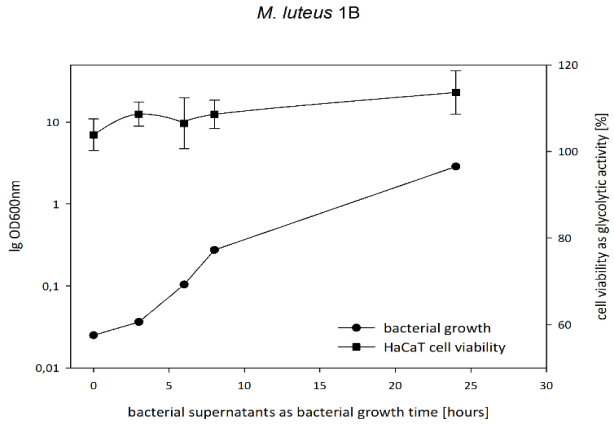


**Fig. S2.** Negative controls for MTT assays with bacterial supernatants from lysogeny broth cultures of *M. luteus* 1D (**a**), *B. licheniformis* 2C (**b**) and *M. luteus* 1B (**c**), respectively. Data shown represent the mean of three biological replicates with p < 0.01.


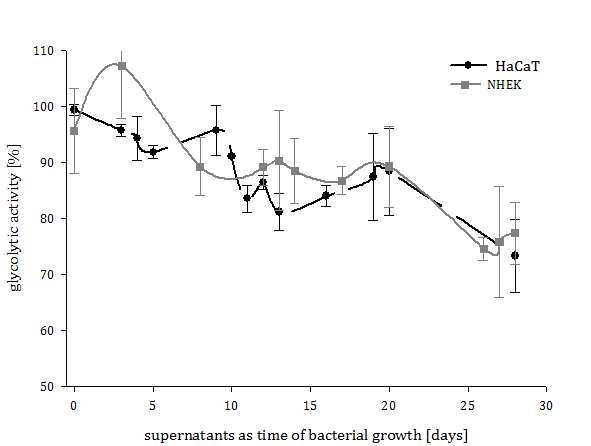


**Fig. S3.** Cytotoxicity of bacterial supernatants excreted by the partial degrader *B. licheniformis* 2C during growth on 100 µM B[*a*]P. Supernatants were added to HaCaT and NHEK cells as indicated and cellular glycolytic activity recorded after 48 h using an MTT assay. Data shown represent the mean of four biological replicates with p < 0.01. Negative controls comprised bacterial minimal medium or supernatants from lysogeny broth cultures.


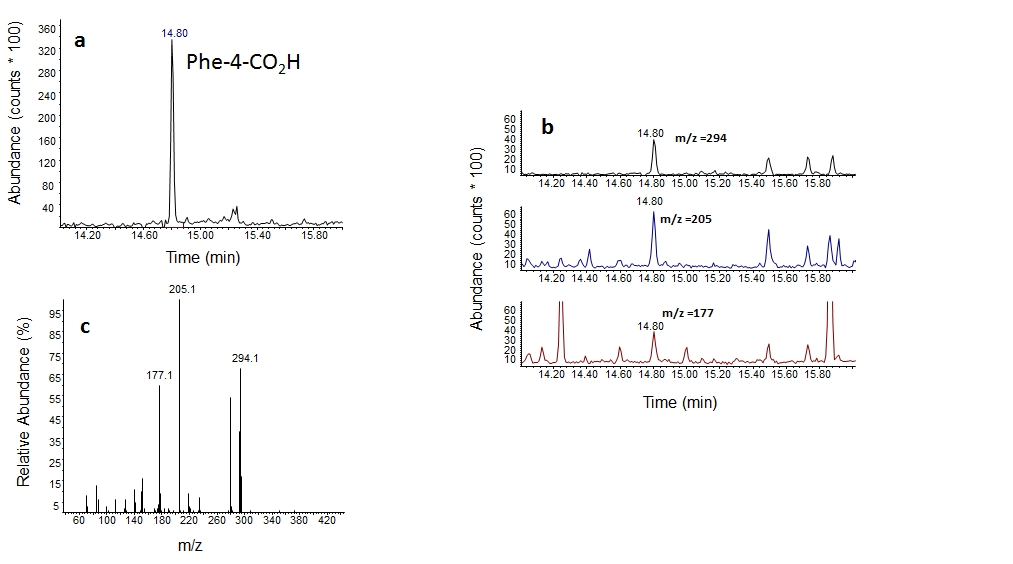


**Fig. S4.** GC-MS ion chromatograms of m/z = 294, m/z = 205 and m/z =177 indicating metabolically formed phenanthrene (Phe)-4-CO_2_H from *M. luteus* 1D. Shown are the chromatograms of the synthetic standard at the retention time of 14.80 min (**a**); together with the metabolites as excreted (most characteristic ion chromatograms, retention time of 14.80 min) (**b**); and the corresponding mass spectrum of the standard compound (**c**).

**Table S1.** Summary of the results of the reverse mutation assay according to Ames. The test was performed using *Salmonella typhimurium* TA95 and TA1535 as well as *Escherichia coli* WP2 *uvrA* suitable for detecting frameshift mutations (*S. typhimurium* TA95) and base-pair substitions (*S. typhimurium* TA1535 and *E. coli* WP2 *uvrA*), respectively. Bacterial supernatants were tested in absence or presence of S9-mix as indicated. Unless stated otherwise test results were considered to be positive once the number of revertant colonies equalled at least three times the count of the corresponding solvent control (induction factor ≥ 3). Data shown represent the mean of three biological replicates. Also tested were the identified metabolites (Table 4) without the addition of S9-mix. However, at their maximally excreted concentrations they did not induce clear reverse mutations, neither did the reconstituted mix.

|  | ***TA98*** | ***TA1535*** | ***WP2 uvrA*** |
| --- | --- | --- | --- |
|  | ***-/+ S9*** | ***-/+ S9*** | ***-/+ S9*** |
| Solvent control (DMSO) | -/- | -/- | -/- |
| Negative control (MM) | -/- | -/- | -/- |
| 2-Nitrofluorene [4 µg/plate] | +/+ |  |  |
| Sodium azide [1.5 and 4 µg/plate] |  | +/+ |  |
| 4-Nitrochinoline-n-oxide [1 µg/plate] |  |  | +/+ |
| Bacterial supernatant *M.luteus* 1D | -/- | -/- | -/- |
| Bacterial supernatant *B.licheniformis* 2C | -/- | -/+ | -/- |
| Bacterial supernatant *M.luteus* 1B | +/+ | +/+ | (+/+)^#^ |

^#^ induction factors of 2.7 and 2.9, respectively
